# COVID-19 lockdown and lifestyles: A narrative review

**DOI:** 10.12688/f1000research.52535.2

**Published:** 2024-09-26

**Authors:** Sathyanarayanan Doraiswamy, Sohaila Cheema, Ahmad Al Mulla, Ravinder Mamtani

**Affiliations:** 1 Institute for Population Health, Weill Cornell Medical College in Qatar, Ar Rayyan, Doha, Qatar; 2 Department of Medicine, Hamad Medical Corporation, Doha, Qatar

**Keywords:** COVID-19, Lifestyle, Diet, Physical activity, Stress, Smoking, Substance, Alcohol, Emotional well-being, Social connectedness

## Abstract

**Background:**

The primary objective worldwide during the coronavirus disease 2019 (COVID-19) pandemic had been controlling disease transmission. However, lockdown measures used to mitigate transmission affected human behavior and altered lifestyles, with a likely impact on chronic non-communicable diseases. More than a year into the pandemic, substantial peer-reviewed literature emerged on altered lifestyles following the varying lockdown measures imposed globally to control the virus spread. We explored the impact of lockdown measures on six lifestyle factors, namely diet, physical activity, sleep, stress, social connectedness, and the use of tobacco, alcohol, or other harmful substances.

**Methods:**

We comprehensively searched PubMed and the World Health Organization’s global literature database on COVID-19 and retrieved 649 relevant articles for the narrative review. A critical interpretative synthesis of the articles was performed.

**Results:**

Most of the articles included in the review identified the negative effect of lockdown measures on each of the lifestyle factors in many parts of the world. Encouraging lifestyle trends were also highlighted in a few articles. Such trends can positively influence the outcome of lifestyle-related chronic diseases, such as obesity and diabetes.

**Conclusions:**

The lockdown associated with COVID-19 has largely had a negative impact on the lifestyles of individuals and communities across many countries and cultures. However, some individuals and communities also initiated positive lifestyle-related behavioral changes. If the knowledge generated by studying the impact of COVID-19-related lockdowns on the six lifestyle factors is further consolidated, it could improve chronic disease outcomes. This will help better understand lifestyle behaviors amidst crises and assist in redesigning extreme public health measures such as lockdowns.. It is up to governments, communities, and healthcare/academic entities to derive benefit from lessons learned from the pandemic, with the ultimate objective of better educating and promoting healthy lifestyles among communities.

## Introduction

The coronavirus disease 2019 (COVID-19) pandemic caused thousands of deaths, overloaded health systems, and disrupted social and economic infrastructure worldwide. While the world remained focused on controlling the pandemic, we cannot ignore its impact on lifestyles and, in turn, on chronic non-communicable diseases (NCDs), which account for over 70% of global mortality. COVID-19 transmission-mitigating measures—requiring people to stay at home and practice physical distancing—afford us the opportunity to examine lifestyle factors and understand their effect on NCDs.

Various degrees of COVID-19 lockdown measures, including travel and movement restrictions, had helped reduce the spread of the virus. The lengths of lockdowns varied from country to country, as gathered from various news articles, ranging from 4 days in Turkey to 300 days in Qatar. The most common pattern of lockdowns was often for shorter periods (2–3 weeks) with extensions as needed. In the United States of America, the range was 20–267 days in different states.

However, these measures also have the potential to diminish access to healthy food; widen inequities between communities; result in suboptimal physical activity levels; exacerbate anxiety and stress; impair the quality of optimum sleep; and encourage substance abuse to allay anxiety and fear of the disease ^1^. Substantial published literature has emerged on altered lifestyles during the pandemic in different populations ^2– 4^; however, to our knowledge, there has not been any synthesis of this literature at this stage of the pandemic.

We conducted an extensive narrative review to help understand lifestyle factors, particularly those related to NCDs, in the COVID-19 context. The review aims to synthesize alterations (positive or negative) in lifestyle factors among individuals and communities, that arose because of varying countries’ lockdown measures. The review can serve as a guidance and a valuable source for healthcare institutions and stakeholders interested in improving healthy lifestyles and healthcare during future lockdown measures. Additionally, it can inspire further research in the emerging field of lifestyle medicine and its role before, during, and after crisis situations, such as pandemics. For the review, we recognize the American College of Lifestyle Medicine (ACLM) definition of a healthy lifestyle as one that includes eating a predominantly plant-based diet, being physically active, sleeping well, managing stress, remaining socially connected, and refraining from the use of tobacco, alcohol, and other harmful substances. Evidence points towards the fact that with optimum practice, these six healthy lifestyle factors can prevent, treat, and, in some cases, even reverse NCDs. This narrative review focuses on these evidence-based lifestyle factors and how they may have been altered during the COVID-19 pandemic. It is to be noted that the authors chose to do a narrative review as this is a less formal approach to a systematic review, as the pandemic was still evolving. This narrative review hopes to raise research questions and identify gaps for future systematic reviews.

## Methods

The narrative review was conducted using a best-evidence synthesis approach put forth by Green *et al.* ^5^. Following this approach, the expectation from a successful narrative review is to: “present information that is written using the required elements for a narrative review, be well structured, synthesize the available evidence about the topic, and convey a clear message” ^5^. Following the Scale for the Assessment of Narrative Review Articles (SANRA) ^6^, the methodology with the checklist is reported as Supplementary Material 1 in the Extended Data ^7^.

## Search strategy

The search strategy included a combination of keywords and controlled vocabulary (such as MeSH terms). The keywords included the following: COVID-19 AND (lifestyle OR dietary habits OR physical activity OR sleep OR tobacco OR alcohol OR substance OR stress OR relationships) AND (lockdown OR quarantine OR isolation). We searched using these terms in PubMed and the World Health Organization (WHO) global literature database on coronavirus disease initially in September 2020 and updated the search in PubMed on 31 January 2021. It is to note that the WHO database is the best available composite database which covers all major databases for COVID-19 publications. These two extensive databases were deemed sufficient by the authors given the narrative nature of the review. We placed no restrictions on language, geography, or publication date. The reference lists of the retrieved articles were also scanned for additional articles with relevance to our review. The number of retrieved items from each database is provided in the Extended data, in Supplementary Material 2 ^7^.

### Inclusion and exclusion criteria

We included all articles that discussed lifestyle factors in the general population within the COVID-19 pandemic context. The review focused on the six lifestyle factors included in the definition of a healthy lifestyle by the American College of Lifestyle Medicine.

Articles were excluded if they did not focus specifically on the six lifestyle factors (dietary habits, physical activity, sleep, tobacco or other substance use, stress, and emotional well-being and social connectedness) or did not discuss lifestyle within the COVID-19 context. We excluded articles that discussed lifestyle change in healthcare workers as they are a specialized group and subject to additional stressors (long work hours, use of personal protective equipment, high risk of contracting COVID-19, dealing with deaths, constant pressure of new admissions, lack of adequate ventilators etc.) beyond the effects of the lockdown measures. Additionally, we excluded published literature reporting modeled effects of lockdown during COVID-19.

### Information extraction

Identified articles meeting the inclusion criteria were imported into Endnote X9 ^8^. Thereafter, the authors screened the title and abstract of the identified articles. Full texts of all relevant articles were further screened for useful data to be eventually included in the critical interpretive synthesis ^9^. From each article, qualitative and quantitative information on the COVID-19-related lockdown and its effects on any of the six lifestyle factors (diet, physical activity, stress, connectedness, sleep, and tobacco or other substance use) on healthy individuals and those with pre-existing NCDs were extracted. In articles that were primary studies with quantitative data, we specifically scrutinized the study design, results, and the conclusion. In articles other than primary studies, we focused on the discussion and conclusion sections. For qualitative studies, opinions/perspectives, and viewpoints, we took a meta ethnographic approach ^10^ as part of our interpretative synthesis. Though the ACLM definition combines smoking, alcohol, and substance use as one lifestyle factor, we reviewed these separately to explore them in greater depth. The information extracted from the articles was classified according to the six lifestyle factors and is summarized in the Results.

## Results

Our search yielded a total of 2620 articles. Following the title, abstract, and full-text screening, 649 articles relevant to the narrative review scope were qualitatively synthesized according to the six lifestyle factors – dietary habits, physical activity, sleep, tobacco or other substance use, stress, and emotional well-being and social connectedness. Though all included articles are reported in the Extended data, in Supplementary Material 3 ^7^, the manuscript bibliography was restricted to an optimal number applying the principle of ‘concept saturation’ ^11^ for several analytical findings.

### Dietary habits

Most of the literature included in the narrative review referring to dietary habits indicated an alteration in the eating behavior of people in many parts of the world during the COVID-19 pandemic ^12, 13^. Most survey-based studies reported an increase in the intake of unhealthy foods by the majority of respondents, a small number highlighted a higher intake of healthy foods, and some studies, such as one from Spain with 1036 participants, reported a wide variation in the type of food (healthy vs unhealthy) consumed by the participants ^14^. In richer households, staying home encouraged excessive use of unhealthy foods, such as processed and fast foods in many parts of the world ^15– 18^. An Italian study reported an increased uptake of ‘comfort foods’ including chocolates and ice-creams used by participants to allay lockdown anxiety ^15^. In a study of 938 French adults during the lockdown, an individual’s mood was a significant factor for food choice ^18^. Similarly, a high prevalence of ‘emotional eating’ was reported in Saudi Arabian women ^19^ and among a cohort of 675 Spanish individuals ^20^. Additionally, some people including children had inclined towards snacking to overcome boredom ^21^,

Staying home also limited access to fresh vegetables and fruits, particularly in the most vulnerable and poorest sections of the society due to limited availability, accessibility, and affordability ^22, 23^. Poorer households were overwhelmed by food insecurity, as demonstrated by a study from Iran reporting 61% of 392 families having faced some level of food insecurity ^24^.

In some situations staying at home encouraged many individuals to eat a fresh, home-made, balanced diet ^25^, as evidenced by a reduction in the consumption of fast food ^26^, fewer purchases of ready-made meals, and by the dominance of online/in-person grocery shopping as the major source of food and food purchases in some countries ^27, 28^. A study from China showed that families that did online shopping included a greater variety of foods in their diet than in normal circumstances ^28^. A large study of 7514 participants from Spain—predominantly young, university-educated females—reported a higher intake of fruits, vegetables, or legumes than during the pre-pandemic times ^29^. Gender (females faring better), family members staying at home, (less) TV watching while eating, and (higher) maternal education were determined to be factors influencing healthy food intake ^30^.

The change in dietary habits during the lockdown period had consequences. One Polish study with 2381 participants reported that 34% of respondents ate more during the lockdown ^31^, while another study from the same country with 1097 participants reported that 30% gained weight during the lockdown ^32^. Meanwhile, among 3533 respondents from Italy, 46.1% reported consuming more food and 19.5% had gained weight ^15^. A similar proportion (46%) of the Galician population from Spain also reported eating more than before the lockdown and 44% of the 1350 adults in the study reported weight gain ^27^. A qualitative study from Ghana found similar eating behavior leading to weight gain during the lockdown ^33^. This weight gain was more pronounced in people with pre-existing overweight/obesity ^34^. Boredom snacking had led to relatively quick excessive weight gain among children ^15, 21, 27, 35– 37^. A Chinese retrospective study of 10,000 studentsalso found an increase in body mass index in most of the participants ^22^.

In the review, we find that individuals who were overweight/obese pre-pandemic gained more weight during the pandemic, while the undernourished lost more weight ^15, 32^. A study in Italy of 88 families found that children with obesity found it difficult to practice a healthy lifestyle during the pandemic, despite participating in a food education program in a hospital. Their unhealthy food consumption and sedentary lifestyle were attributed to a great deal of stress experienced by the children due to interruption of school and sports activities among other factors ^38^. Studies also reported that individuals with eating disorders and other psychiatric disorders diagnosed prior to the lockdown fared worse during the lockdown period due to low self-directedness and less adaptive coping ^17, 39– 44^

### Physical activity

The lockdown period saw a general reduction in physical activity levels as evidenced from the studies included in the review. A study from China of 339 participants revealed a significant reduction in the average exercise intensity for both males and females during the lockdown period (average steps per day, before vs during: 7038±1923 vs 3741±1042 steps, p < 0.001 for females and 8321±3000 vs 3728±1726 step, p < 0.001 for males) ^41^. A similar observation was made in Italy where 68% of participants in a study with a sample size of 490 adults reported a decrease in exercise levels ^42^. In a Polish study sample of 238, 43% of participants also reported a reduction in their physical activity during the lockdown period ^32^. An international, multi-country online survey of 1047 participants found that the number of days per week of all physical activity declined by 24% during the lockdown period. The number of minutes per day spent on physical activity decreased by 33.5% while sitting time increased by 5–8 hours among the respondents ^43^. Diminished physical activity levels were also reported in other countries, such as Australia ^44^, United Kingdom (UK) ^45^, and the United States (US) ^46, 47^. Longitudinal tracking of steps among participants in a physical activity program in Australia saw a decrease in the number of steps during lockdown, which returned to normal with the lifting of restrictions ^48^. A similar improvement in physical activity levels was observed after the end of lockdown in Greece ^49^.

The work-from-home environment predisposed individuals to spend more time sitting, leaning, reclining, or lying down on the bed ^17^. However, there was no predictable pattern as to who was less active during the lockdown period. While some studies found that previously physically active individuals became less active during the lockdown period ^47, 50^, other studies found no change ^46^ or reported a further reduction in physical activity among previously inactive individuals ^50^. A cross-sectional study in 64 cities in China, with 369 participants, concluded that previously physically active individuals were in greater distress because of the lockdown ^51^. Studies among preschoolers ^52^, children and adolescents in Spain ^53^ and Brazil found a downward trend in physical activity levels and an increase in screen time/sedentary behavior among children during the lockdown period ^4^. A similar reduction in physical activity levels among children and adolescents was also reported in studies from Canada, Germany, UK, Italy, and Spain ^54– 59^. A study in India found a reduction in physical activity and increased screen time among men and those belonging to the upper socio-economic strata ^60^. Ironically, a study from Germany found that participants who regularly consumed alcohol and expressed greater satisfaction with their life were more physically active ^61^. It was also observed that older people who were single/alone ^62^, affected by frailty ^63^, used to exercising in groups, or who were not accustomed to online apps (which support exercise routines) experienced a reduction in physical activity and/or increased levels of sedentary behavior ^64– 66^.

Lockdown provided an opportunity to be creative in finding ways to stay physically active. In South Korea, for example, it was observed that the public bicycle-sharing system recorded an increase in commuter and weekend users ^67^. It is, however, to be noted that such initiatives required commitment from citizens and authorities to ensure adherence to physical distancing, hand hygiene, facemask use, and cleaning of public facility/site surfaces. Staying physically active in home environments was possible, and its feasibility was shown during COVID-19 lockdown periods in countries like Japan ^68^, Scotland ^69^, and Switzerland ^70^, particularly among children with good parental support and encouragement. A study of 213 university students in Spain found an increase in the mean number of days they were engaged in moderate and vigorous physical activity ^71^. The use of physical activity apps was useful in improving physical activity in the US ^47^. Another study of 1521 adults from the UK reported that, although most adults claimed to have maintained or increased their engagement in physical activity during the pandemic, the majority did not meet the recommended physical activity guidelines ^72^. Studies performed in several countries, including a large study of 43,995 individuals from Brazil ^73^, reconfirmed that individuals who were physically active during the lockdown period were more resilient and had lower odds of suffering from stress, anxiety, and depression ^74– 83^.

### Sleep

Stay-at-home restrictions affected the sleep of people in many parts of the world ^84, 85^. A global study involving 6882 participants from 59 countries found a large proportion (66.2%) reported changes in their sleep patterns during the pandemic ^86^. In a large cross-sectional study across France, two weeks after the lockdown, 74% of the 1005 nationally representative participants reported problems trying to sleep as compared to 49% in a previous survey pre-lockdown ^87^. An Italian study including 400 university students and administrative staff reported that 40% of the respondents suffered from insomnia, while 15% specifically had issues in sleep initiation ^88^. A similar proportion (40.7%) among a sample of 1491 adults in Australia reported a negative change in sleep levels ^44^. Factors found to be associated with disturbed sleep were increased blue light exposure from extended screen time, browsing the internet to keep busy, and working remotely from home ^88, 89^. Older age, living with a partner, and living in a higher income country were associated with better sleep health, while loss of job, challenges experienced in transitioning to working from home, being unable to pay important bills, and an increase in arguments and conflicts at home were associated with poorer sleep health ^90^. Poor sleep and anxiety/depression were found to be closely associated in a few studies ^91– 93^.

Respondents in some studies reported sleeping more during the lockdown period. This was particularly true for preschoolers, children, and adolescents, as observed in studies from Brazil, Spain, Canada, and China ^4, 17, 54, 55, 71^. Among adults who slept more, the reported sleep quality was poor ^86, 94^. Those who slept more also experienced sleepiness during the day and increased daytime sleep duration, as reported in a study of 203 corporate professionals and 325 university students in India ^95^. In the same study, it was also observed that females, younger people, and those experiencing loneliness and COVID-19-related worries had greater difficulty with sleep ^95^.

Frequent awakening and increased sleeping difficulty were noted in 69.6% of 365 patients in Portugal who had pre-existing sleep-disordered breathing ^96^. One study reported an increased frequency in the uptake of sleeping pills among study participants ^87^. Increased stress due to COVID-19, information overload, inconsistent bedtime, and low mood contribute to insomnia and this has subsequent harmful effects on overall health ^96^.

A few studies reported that individuals with previously erratic sleep patterns and social jetlag, were provided with an opportunity to normalize their sleep patterns and improve sleep hygiene for optimum functioning ^94, 97– 100^. One study from the US found that reduced rigid workload and school schedules led to an improvement in the overall sleep quality for some individuals ^101^, and a study in Canada found students were better able to sleep and reported reduced daytime sleepiness during the lockdown ^102^. A beneficial impact of quarantine on sleep was documented by a large global study of 14,000 individuals from 11 countries. A very small proportion of people were found to suffer from anxiety-induced insomnia in this study ^103^. At least one study showed that sleep problems encountered post-lockdown declined over time ^90^.

### Substance use

***Tobacco.*** Few studies were available on smoking behavior during COVID-19 lockdowns. Among those available, we found variations (increases, decreases, and remaining at pre-lockdown levels) in the smoking patterns among the respondents ^104^. A cross-sectional study involving 345 participants in the US found that half of respondents retained their pre-lockdown level of smoking, one quarter of them reduced smoking, and the remainder increased smoking ^105^. The same study also found that while 20% of respondents showed an increased motivation to quit, 15% showed a reduction in motivation ^105^. In a study of 957 participants from the Netherlands, 14.1% reported smoking less during the pandemic, while 18.9% reported smoking more ^106^. In Australia, 6.9% of 1491 adults reported a negative change in their smoking habits after the introduction of lockdown measures ^44^. In a multi-country study involving 6800 combustible and e-cigarette users from Italy, India, South Africa, UK, and the US, indoor smoking had increased in India and Italy, thus increasing the risk of secondhand smoke exposure among family members ^107^. Anxiety, boredom, and stress experienced by many people during COVID-19 contributed to increased smoking in some individuals ^27, 104, 108, 109^. Stress and inactivity were cited as the main reasons for increased smoking during lockdown by a cohort of patients with cardiovascular disease in France ^110^.

Despite many deterrents, motivated individuals saw the lockdown period as an opportunity to become healthy and quit smoking ^105^. The motivation to quit was more discernible among those who smoked tobacco cigarettes than electronic cigarettes ^105^. Smokers who had a family member or someone close to them suffer from COVID-19 displayed a greater motivation to quit ^105^. One study reported that many parents wanted to appear on their best behavior in front of their children during lockdown and postulated that this was one of the reasons for them cutting down on their cigarette intake when spending more time at home ^111^. In a qualitative study in the US, several participants mentioned that their cessation efforts improved due to a decrease in opportunities for social smoking and because they were spending more time at home. However, other individuals in the same study claimed an increase in smoking due to lack of the same restrictions at home compared to those imposed at their workplaces ^104^.

***Other harmful substances.*** Among other substances that cause dependence, alcohol stands out as one of the most used psychoactive substances during the lockdown period ^108, 112– 114^, despite alcohol supply not being assured in many countries. A study from India documented that alcohol shop closures led to alcohol-dependent individuals experiencing withdrawal symptoms and suicides ^115^. Where alcohol was available, some people resorted to increased alcohol intake to overcome adverse psychological outcomes of the lockdown, including stress and insomnia ^116, 117^. Data from the Office for National Statistics in the UK demonstrates that alcohol sales rose by over 30% in month-on-month volume when compared to the previous year. In a survey of 6416 people from China, 32% of regular alcohol drinkers reported increased consumption during the lockdown period ^118^. In Australia, 26.6% of 1491 participants reported an increase in alcohol consumption during the lockdown ^44^. A higher frequency of drinking was selectively observed in men, young adults aged 18–24 years, and those living in big cities ^119^. A study in Italy observed that emergency admission of adolescents and young people in one hospital due to alcohol-related emergencies increased manifold during the lockdown ^56^. In a survey of 1555 people in the UK, one third reported that they had stopped or reduced drinking during the lockdown, while one fifth drank more frequently during the same period ^120^. A survey of 4072 participants in Poland found a reduced frequency of drinking in most participants ^119^.

It was identified that those who consume alcohol found it more difficult to cope with the mental health impact of the pandemic and associated lockdowns ^113^. A large study from Australia found that people who were heavy drinkers, middle-aged, and had higher incomes before the pandemic, increased drinking during the pandemic ^121^. This study also found that individuals who had experienced recent job loss, were stressed/depressed, or had altered eating and sleep habits consumed larger amounts of alcohol than those who weren’t ^122^. A study from the US found an increase in harmful alcohol use and related behaviors over the six months of lockdown-related measures ^123^.

A survey of 1555 patients with pre-existing alcoholic disorders in the UK concluded that ‘lockdown represents a risk factor for increased alcohol consumption in people with alcohol use disorders and relapse for those who were previously abstinent’ ^124^. Individuals who relapsed were at a high risk of harmful drinking and required a custom-tailored approach for follow-up and intervention.

A study of university students before and after university closures in the US revealed that those students who moved from on campus accommodation with peers to their homes with parents demonstrated a significant reduction in their drinking than those who remained in their pre-pandemic locations ^125^. A study of 1951 students from Belgium found that pre-lockdown drinking motives reliably predicted alcohol consumption during the lockdown ^126^.

With regard to addictive drugs, the European Monitoring Centre for Drugs and Drug Addiction determined that people using addictive drugs may be at higher risk during the crisis, particularly because of the aging cohort of opioid users and associated comorbidities. Experts also agreed that movement restrictions reduced the availability of illicit drugs ^127^, thereby increasing the demand for addiction treatment. The limited availability of these services added to the suffering of this population. There was one study from France, however, which found an increase in cannabis use among 31.2% (195 of 620) of regular cannabis users making up 5.44% (620 of 11,391) of total participants ^128^. Another study from the Netherlands found increased use of cannabis among daily cannabis users ^129^. In addition, studies have demonstrated high anxiety levels among pre-COVID-19 substance users and COVID-19 substance initiators ^130^.

### Stress

Watching, reading, or listening to the constant stream of COVID-19-related news, perceived health risks ^131^, and a perceived lack of autonomy ^132^ led to increased anxiety among individuals and families in many countries ^133– 138^. The 2020 National Health Interview survey found that US adults were eight times more likely to fit the criteria for serious mental distress during the lockdown as compared to before ^139^. A study from China reported the psychological impacts of COVID-19, expressed as mild-to-severe anxiety, among 25% of 7143 college students ^140^. In total, 65% of 432 respondents in another study from Hong Kong reported clinical levels of depression, anxiety, and/or stress. Older adults, especially those in isolation and with cognitive decline ^141, 142^, and their caregivers ^143^ were identified to be more prone to anxiety, stress, and depression ^133^. A high prevalence of stress and post-traumatic stress disorders was documented among Nepalese ^144^, Lebanese ^145^, Saudi Arabian ^146, 147^, Vietnamese ^148^, and Philippine nationals ^147^ at the height of lockdown. High levels of anxiety and depression were also documented in studies from India ^149– 151^, Ecuador ^152^, Albania ^153^, Ireland ^154^, Hungary ^83^, Spain ^155^, Palestine ^156^, the UK ^157^, Kuwait ^158^, Brazil, and Portugal ^159^. The prevalence of people reporting clinical depression and anxiety in a sample of 1215 respondents in Italy was recorded to be 32.3% and 35.7%, respectively (compared to 15.39% and 21.40%, respectively, during pre-COVID-19 times) ^136^. A similar finding was made by another study from the country ^160^. One study from Jordan of 5274 participants found that four out of every ten participants in home quarantine suffered from some degree of anxiety ^161^.

Articles also suggest that quarantined children ^162^, adolescents ^163^, university students ^164– 166^ and adults ^167^, mothers of young children ^168^, and other healthy adults suffered from symptoms of poor mental health, such as post-traumatic stress, anger, and confusion ^169, 170^. One study in Turkey found a difference in the self-reported quality of life (better) of children as opposed to the perception of their parents ^171^. Only a few studies made a clear distinction between the stress levels experienced by the general population as part of the community quarantine versus those who were quarantined as part of contact tracing or after testing COVID-19 positive. A few studies seem to suggest greater stress experienced by both those under isolation ^172, 173^ and others under home quarantine as part of contact tracing ^174^. One study from China, however, found that home self-quarantine was associated with increased happiness as compared to those who were in community quarantine ^175^. A study from the UK found that a majority of the older people interviewed identified positive aspects of lockdown and felt better prepared to deal with the lockdown ^176^.

Higher stress levels associated with lockdown measures were observed in some groups, such as women ^177– 180^ (particularly pregnant women ^181^, single/divorced adults ^76^, older people (particularly with pre-existing mental health conditions ^154, 167^), parents with young children ^182^, university students and young adults ^183– 185^, workers ^186^, those self-medicating ^187^, the economically deprived ^137^, those with pre-existing depression ^188^, bipolar disorders ^189^, and other chronic medical conditions ^133, 180, 190– 192^. A large study of 56,679 participants in China found that quarantine had a profound effect on the mental health of specific vulnerable groups, including those with pre-existing mental disorders, chronic physical diseases, frontline workers, and those living in the most affected areas ^193^. A similar large study of 53,524 participants from 26 countries during the pandemic found higher levels of stress associated with being young, female gender, lower level of education, being single, number of children in the family, and living in a country experiencing severe community spread ^194^.

Extreme stress driven by fear of COVID-19 infection, financial crisis, loneliness, the pressure to quarantine and isolate when tested positive, stigma, and the unavailability of alcohol were identified as drivers of suicidal ideation and suicide during the pandemic and the associated lockdowns ^173, 195– 197^. A healthy diet and refraining from reading updates about COVID-19 were very often shown to be the best predictors of lower levels of anxiety and depressive symptoms ^198^. A longitudinal study reported that fear of COVID-19 decreased over time since the introduction of lockdown, but depression levels increased in a cohort of Chinese individuals ^189^. There were mixed results from New Zealand, one of the countries known to have best managed the pandemic, where 62% of those surveyed in a study reported ‘silver linings’ during the lockdown, such as enjoying working from home and a less polluted environment, while the same study found 16% of participants suffered from anxiety and 39% reported low well-being ^199^. A qualitative study from Italy found that, beyond the difficulties associated with lockdown, parents of children with neurodevelopmental disorders appreciated spending more quality time with their children, thus contributing to enhanced parent-child relationships ^200^. Some longitudinal studies found the mental health of individuals improved over time as extreme fear and the sense of emergency subsided ^201^.

### Emotional wellbeing and social connectedness

The restriction of movement during the pandemic led to a significant reduction in social activity among family, friends/neighbors, and for entertainment ^202, 203^. This contributed to diminished life satisfaction in >30% of participants in a large, multi-continent study involving 1047 participants ^203^. A study of 571 healthy adults in Israel found greater psychological distress, poor connectedness, and lowered resilience among women, young adults, and the unemployed ^204^. An online survey of 309 residents in Europe and the US revealed that 100% of participants faced some degree of social isolation during the lockdown period. This study found that younger adults (aged 18–29) reported higher self-isolation than older groups. Those feeling socially isolated were also less satisfied with their food habits, work, and housing ^205^. Prolonged social media use had a deleterious effect on the overall social well being of individuals as reported by studies in Saudi Arabia ^202^ and Italy ^206^.

A study in Finland ^207^ found that families used two types of coping during the lockdown – family conversations and family time. Family time, defined as “doing things together,” was more prevalent on weekends, when families preferred to do outdoor activities, such as playing together A study in Canada ^208^ found that time spent on the internet and video games by children increased during lockdown periods. No studies during the review period explicitly documented changes in other positive indoor behaviors, such as yoga and meditation. 

Studies found that a lack of social interaction had a more profound effect on people with higher income and education ^204^. Lack of social interaction also had a negative impact as more time passes for older people, as evidenced by large cross-sectional studies in the UK ^209, 210^ and Switzerland ^211^. Children and adolescents reported being more restless, irritable, and inattentive during the lockdown ^212^ with increased screen time being the likely contributor ^213, 214^ along with worsening of relationships ^215^ and dysfunctional parenting ^216^. Girls and women confined to homes and quarantine facilities faced increased family tensions, an overload of domestic work, and were vulnerable to physical and emotional abuse ^217^. Intimate partner violence—characterized by physical, sexual, or emotional violence between partners—was also of concern during challenging times of staying home and with physical distancing ^218^. A study in Switzerland found that older adults who managed to maintain social communication at a satisfactory level were able to manage their stress level, thus underscoring the importance of social connectedness ^211^.

### Lifestyle factors in patients with pre-existing NCDs

Studies show that patients with pre-existing NCDs found it difficult to maintain a healthy lifestyle during the COVID-19 pandemic and associated lockdown measures introduced by countries ^219^. Patients with coronary heart disease ^220– 222^, neurological disorders ^223, 224^, diabetes ^225, 226^ and cancer ^227, 228^ had to compromise on their physical activity routine, dietary patterns, and social connectedness in particular ^219, 226, 229, 230^, as did families with children who had chronic respiratory diseases ^184, 231^. Poor glycemic control in diabetics due to altered lifestyle was reported in many studies ^232– 238^, while one study in India reported an improvement in the glycemic status of patients with type II diabetes mellitus ^239^. In a study from France, 45% of 195 patients with chronic heart disease had a >25% reduction in physical activity and 24% of patients had gained >2kg weight ^240^. A study of parents of 125 children with disabilities in the UK found that 61% of them reporting a reduction in physical activity levels and over 90% reported a negative impact on their children’s mental health ^241^. A qualitative study in Ireland came to a similar conclusion regarding increased mental health difficulties among children with Autistic Spectrum Disorder ^242, 243^. Patients fearful of acquiring COVID-19 infection were also found to postpone medical care for their chronic problems ^244^, leading to greater physical and psychological distress, while adding to the suffering of chronically ill patients ^219, 245, 246^. Some studies found an improvement in patients with conditions such as migraine ^247, 248^, whereas other studies found limited or no change in the lifestyle of those with other chronic diseases ^249^.

## Discussion

Our narrative review highlights the altered lifestyles of people worldwide during the lockdown period of the pandemic. Both positive and negative changes in the six lifestyle factors have occurred and their impact varied in different instances. These findings could be instructive to health practitioners and health departments in their strategies to manage chronic disease.

Regarding dietary habits, at the start of the pandemic the United Nations Systems Standing Committee on Nutrition warned that the COVID-19 pandemic would disrupt food systems globally. The committee predicted a deterioration in the nutritional status of individuals due to altered dietary practices ^250^. Particular concern was expressed over ‘food environments’, with regard to both external (availability and quality) and internal (accessibility, affordability, acceptability, and desirability) dimensions of the food systems ^250^. Observations, more than a year after the onset of the pandemic, now appear to confirm earlier fears of malnutrition and disrupted food environments, particularly in disadvantaged communities ^251^. In general, the availability of healthy, plant-based food was ample in well-to-do communities. However, access to such food during the pandemic could be limited, at least in some settings and for specific populations. This lack of access may present health risks, such as lower immunity and adverse chronic disease outcomes, to individuals in those settings. Alternatively, staying at home encouraged individuals in some populations to eat fresh, homemade food ^252^, likely due to additional time available to cook. Home-cooking not only helps cut down the intake of unhealthy processed and fast foods but also has proven emotional and health benefits. Further research is needed to understand the motivational factors behind healthy dietary practices during the pandemic and whether pre-pandemic awareness and practices likely had a positive influence.

It is well known that any amount of physical activity, no matter how little, is beneficial. Sedentary behavior associated with excessive television watching, increased internet browsing, playing screen games, and using mobile devices are shown to negatively impact chronic disease outcomes ^253^. With parks and gymnasiums closed and public movement in the open discouraged, people’s ability to remain physically active was challenged during the lockdown period. Though the majority of studies showed a reduction in physical activity levels and increased sedentariness during the lockdown, it is heartening to note that some individuals in select settings saw an opportunity to be creative in finding ways of staying even minimally active (e.g., gardening, exploring online free exercise routines, and walking up and down the stairs/steps) in a home environment ^64, 254^.

Adequate sleep improves concentration and diligent decision-making. Disrupted and suboptimal sleep on the other hand has been correlated with an elevated body mass index, obesity, metabolic syndrome, and type 2 diabetes ^255^. Exposure to blue light also increases night-time heart rate, blood pressure, and core body temperature, and suppresses melatonin and sleepiness. The high prevalence of insomnia and an increase in screen time during the lockdown periods were concerning. Reversal of some of these newfound habits will be important in order to promote sleep hygiene in communities and reap the benefits of sleep in improving mood, immunity, and overall health and well-being of individuals and communities.

Reports of people hoarding cigarettes and alcohol along with food and toilet paper during the early phases of the pandemic are suggestive of the fact that smoking cessation and quitting alcohol was not a priority during the lockdown period; our review confirms this. Some smokers wanting to quit may have found accessing smoking cessation clinics a challenge due to the suspension of non-essential health services., This lack of access to addiction clinics will also affect those dependent on alcohol and other psychoactive substances. Getting substance cessation and control programs back on track at local, national, and international levels will require a redoubling of efforts as countries start relaxing lockdown measures.

Most of the studies in our review have unequivocally pointed towards high stress levels among populations worldwide during COVID-19 lockdowns. Stress is causally associated with risky behaviors, such as smoking, physical inactivity, and heavy alcohol use, lending to additional concern since these are independent risk factors for chronic diseases. However, on the positive side, staying at home provideed an opportunity for families to bond and engage in activities at their convenience, which can elicit relaxation and calming responses. Examples of such activities include prayers, meditation, and breathing exercises, all having positive health benefits ^256^. Nevertheless, we did not find any studies where communities claimed to be using such positive coping measures to alleviate stress. This may indicate a lack of awareness in communities regarding the importance of healthy lifestyle measures for stress management, as well as public health professionals/healthcare practitioners not emphasizing the importance of addressing lifestyle-related factors adequately.

More importantly, one cannot deny that of all the lifestyle factors altered during COVID-19 lockdowns, the most crucial one was the loss of connectedness and social networking. This is unfortunate since positive resonance and micro-moments of connectivity encountered in situations of togetherness are associated with health and longevity. Virtual connectivity, though inadequately studied, and small acts of kindness where possible during this period, remain the best measures of improving relationships and connectedness. The lockdown no doubt led to an increase in family time, at least for nuclear families in most instances. This may have been an opportune time for families to bond and strengthen relationships. One concern of staying home, however, were reports of increased domestic violence ^257^. Future research is likely to provide more answers to the questions surrounding the nature of relationships and domestic activities, and their overall impact—positive or negative—on mental and physical health.

During this period, patients with pre-existing NCDs suffered both from their inability to maintain a healthy lifestyle and neglect from national health authorities and hospitals with guidance issued to delay routine care and non-urgent procedures. Since the advisory by the US Medicare and Medicaid Centers in mid-March 2020 to delay non-urgent procedures, adult patient visits for primary care and gastroenterology services declined by 49% and 61%, respectively ^258^. This likely compromises patients receiving timely care. Tapper and Asrani ^259^ discuss care in the context of managing patients with liver cirrhosis (a common complication of obesity and diabetes) during COVID-19 and suggest three challenging phases in treating patients with chronic disease: 1) the current ‘intense period’ with a delay in routine care, 2) a ‘return to the norm’ when physical distancing ends and health systems respond to a backlog of cases, and 3) a ‘protracted period’ of poor outcomes due to unabated disease progression. The world is headed towards a catch-up period concerning NCDs due to altered lifestyles and concomitant treatment backlog. The emergence of technology, including machine learning and artificial intelligence, offers hope for the future control of both communicable and non-communicable diseases ^260, 261^. 

The American College of Lifestyle Medicine proposes healthy lifestyle measures to reduce chronic disease risk and improve immunity during this period ^1^ ( Table 1). Though the measures may appear relatively easy and simple enough to implement, many people may not adhere to these recommendations for a variety of reasons, including poverty, lack of education, unaffordability of technological resources, cramped living spaces, and poor city planning. Governments, health care practitioners, and communities should do everything possible to break those barriers and ensure access to healthy lifestyle measures for all.

**Table 1.** Healthy lifestyle measures to reduce chronic disease risk and improve immunity ^1^.

• Stress management by practicing techniques like meditation and yoga. • Healthy eating focused on a plant-based diet and whole grains. • Remaining physically active. • Quitting smoking and harmful substances. • Optimizing good sleep hygiene. • Maintaining social connections virtually, like video or phone calls with friends and families.

### Strengths and limitations

This narrative review is a comprehensive analysis of the existing literature on the impact of COVID-19-related lockdown on the lifestyles of people globally. As the review is narrative, we included all the relevant and available published literature on the subject to ensure comprehensiveness. We did not undertake quality assessment of each of the publications; instead, we used data as reported in the articles. Most of the studies included in our review are observational, which is an inherent limitation of the review study design. Despite this general limitation—as is the case with all narrative reviews—we do not consider this a major impediment to our research pursuit, as our analysis relies largely on trends and qualitative aspects of data. Given the global nature of the review, we also acknowledge that the studies included are from various countries and cultures and, hence, cannot be generalized. We have identified patterns, where possible, and we hope, will have broad global implications and specific local implications. We only searched two databases for the study and with the huge volume of studies in this broad subject, it is possible we might have inadvertently missed some studies in our synthesis. The point of concept saturation was based on judgement of the authors though acceptable in an interpretivist paradigm, this can be seen as a limitation in a positivist paradigm. We also acknowledge that home quarantine, institutional quarantine, community quarantine, and isolation can each have different levels of impact on lifestyles, but did not find an adequate number of studies in each category to analyze them separately.

## Conclusions

COVID-19 disrupted lifestyle balance among populations. The lockdown associated with COVID-19 largely had a negative impact on the lifestyles of individuals and communities across countries and cultures. However, some individuals and communities also initiated positive lifestyle-related behavioral change. If the knowledge generated by studying the impact of COVID-19-related lockdowns on the six lifestyle factors is further consolidated, it could improve chronic disease outcomes. This will help better understand lifestyle behaviors amidst crises and assist in redesigning extreme public health measures such as lockdowns.

Based on its rapid assessment of service delivery for NCDs in 163 countries, the World Health Organization had identified that the COVID-19 pandemic could throw the global health progress on NCDs off track. This requires that we act as one world and make every effort to promote healthy lifestyles in communities worldwide. COVID-19 will likely redefine conventional public health by placing additional emphasis on healthy lifestyles, disease prevention, and self-care. Given the strong evidence in favor of lifestyle medicine, the incorporation of lifestyle medicine in day-to-day medical practice and public health approaches is likely to receive worldwide attention. Serious consideration should be given to augmenting professional health curricula by including topics such as evidence-based public health practices, lifestyle medicine, and building health systems that can address the holistic needs of local populations before, during, and after crises. Also, integrating basic health, infectious disease, and lifestyle education in school and undergraduate college education would be a step in the right direction. It would be up to the governments, communities, and healthcare delivery systems to learn and benefit from the lifestyle medicine lessons learnt—during and consequent to the COVID-19 pandemic—with the ultimate objective of promoting healthy lifestyles in communities.

## Data Availability

All data underlying the results are available as part of the article and no additional source data are required. Harvard Dataverse: COVID-19 and lifestyle - a narrative review - supplementary material. https://doi.org/10.7910/DVN/ZIA8LI ^4^ This project contains the following extended data: Supplementary Materials.docx (Supplementary material 1: SANRA—a scale for the quality assessment of narrative review articles; Supplementary material 2: Search results and Supplementary Material 3: Included Studies (2021-04-24)) Data are available under the terms of the Creative Commons Zero "No rights reserved" data waiver (CC0 1.0 Public domain dedication).
