# Supplementary material for: COVID-19 lockdown and lifestyles: A narrative review
Source: F1000Res. 2024 Sep 26;10:363. Originally published 2021 May 10. [Version 2] doi: 10.12688/f1000research.52535.2 (PMC11472275; doi:10.12688/f1000research.52535.2)
Supplement: Peer review history

# Reviewer report: Reviewer response for version 2

**DOI:** 10.5256/f1000research.169755.r327131

**Published:** 2024-10-10

**Authors:** Cavid Baba

**Affiliations:** 1 Institute of Health Scienes, Dokuz Eylül University School of Medicine, Izmir, Turkey; 2 Neurology, Urla State Hospital, Izmir, Turkey

No further comments

Is the work clearly and accurately presented and does it cite the current literature?

No

If applicable, is the statistical analysis and its interpretation appropriate?

Not applicable

Are all the source data underlying the results available to ensure full reproducibility?

Yes

Is the study design appropriate and is the work technically sound?

No

Are the conclusions drawn adequately supported by the results?

No

Are sufficient details of methods and analysis provided to allow replication by others?

Yes

Reviewer Expertise:

neurology, multiple sclerosis, cognition, rehabilitation

I confirm that I have read this submission and believe that I have an appropriate level of expertise to confirm that it is of an acceptable scientific standard.

# Reviewer report: Reviewer response for version 1

**DOI:** 10.5256/f1000research.55830.r291270

**Published:** 2024-07-25

**Authors:** Cavid Baba

**Affiliations:** 1 Institute of Health Scienes, Dokuz Eylül University School of Medicine, Izmir, Turkey; 2 Neurology, Urla State Hospital, Izmir, Turkey

This is a narrative review looking at the lifestyle changes during the COVID-19 lockdown. The researchers used the American College of Lifestyle Medicine (ACLM) definition of a healthy lifestyle with six parameters. I appreciate the researchers' effort to increase the value of the narrative review by using a best-evidence synthesis approach referenced in the paper. However, my main concern is the timing of the publication, as the search was done more than three years ago.

 The following are the questions that drew my attention:

  

1. The manuscript's title suggests a focus on the COVID-19 pandemic. However, in several sections of the text, the researchers focused on modifications and preventive measures during times of crisis, such as lockdown. I would advise that researchers be more detailed in their interpretations of the outcomes. Because the reasons of a crisis or lockdown can vary, so can the effects. Either modify the manuscript's name or make your interpretations totally relevant to COVID-19, or at least to the lockdown during highly transmissive and fatal respiratory diseases.
2. In the introduction section, researchers use the statement "The review can be instructive…" - a narrative review can assist grasp the topic and provide hints, but it is still not regarded a method of high evidence, therefore the term is not acceptable. 
3. In the introduction, the purpose of the review was presented as understanding of lifestyle factors and how they may have altered during COVID-19. However, the discussion and conclusion focused on ways to improve healthy lifestyle and preventive measures to protect that habit. I recommend that researchers focus on the purpose of the review and let the community decide the actions.
4. The search dates cover articles published from September 2020 to January 31, 2021, which is too short to include all relevant and important research, especially since articles related to COVID-19 are still being published. This is a significant drawback for review articles, which should be published in a timely manner to maintain their value. Another option is to extend the search and re-write the article.
5. The researchers removed papers unrelated to COVID-19. As previously noted, if the issue is lockdown during a transmissible disease, the other papers should have been added. Otherwise, terminology and expressions throughout the document should be specific to COVID-19.
6. Why were articles that examined the same factors among healthcare workers excluded? What do researchers mean when they state "additional stressors beyond the effects of the lockdown measures"? If they are more susceptible to contracting the virus, delivery and transportation workers are also at risk. If it is anything else, please provide a reasonable explanation as to why the pressures of other professions, in addition to the consequences of the lockdown, are not regarded significant enough to eliminate from the search. The review's goal is to examine how people's lifestyles changed during the pandemic. I don't see why the researchers made such a difference.
7. The researchers created statements by merging articles from various geographical and cultural backgrounds. Dietary choices and physical exercise, for example, can vary. What is normal in one culture may be unhealthy in another, therefore the changes observed during the lockdown are not proportional.  I'm not sure if such a global mix of specific lifestyle behaviors is fair for comparison.

Is the work clearly and accurately presented and does it cite the current literature?

No

If applicable, is the statistical analysis and its interpretation appropriate?

Not applicable

Are all the source data underlying the results available to ensure full reproducibility?

Yes

Is the study design appropriate and is the work technically sound?

No

Are the conclusions drawn adequately supported by the results?

No

Are sufficient details of methods and analysis provided to allow replication by others?

Yes

Reviewer Expertise:

neurology, multiple sclerosis, cognition, rehabilitation

I confirm that I have read this submission and believe that I have an appropriate level of expertise to state that I do not consider it to be of an acceptable scientific standard, for reasons outlined above.

# Response

**Published:** 2024-09-22

**Authors:** Sathyanarayanan Doraiswamy

The authors would like to thank the reviewer for the comments. Our point-by-point response is as below:

1. The manuscript's title suggests a focus on the COVID-19 pandemic. However, in several sections of the text, the researchers focused on modifications and preventive measures during times of crisis, such as lockdown. I would advise that researchers be more detailed in their interpretations of the outcomes. Because the reasons of a crisis or lockdown can vary, so can the effects. Either modify the manuscript's name or make your interpretations totally relevant to COVID-19, or at least to the lockdown during highly transmissive and fatal respiratory diseases.

**Author Response: The review’s objective is to explore the impact of COVID-19-related lockdown measures on six lifestyle factors: diet, physical activity, sleep, stress, social connectedness, and the use of tobacco, alcohol, or other harmful substances. This has now been made explicit in the introduction in line with the reviewer’s kind suggestion.**

1. In the introduction section, researchers use the statement "The review can be instructive…" - a narrative review can assist grasp the topic and provide hints, but it is still not regarded a method of high evidence, therefore the term is not acceptable. 

**Author Response: This has now been altered to reflect the guiding nature of a narrative review.**

1. In the introduction, the purpose of the review was presented as understanding of lifestyle factors and how they may have altered during COVID-19. However, the discussion and conclusion focused on ways to improve healthy lifestyle and preventive measures to protect that habit. I recommend that researchers focus on the purpose of the review and let the community decide the actions.

**Author Response: The conclusion has now been altered. The section on conclusion also includes recommendations from the findings of the review for a variety of stakeholders including communities to consider. Authors have ensured that all recommendations are evidence based and have also called for further research when available evidence is inadequate. **

1. The search dates cover articles published from September 2020 to January 31, 2021, which is too short to include all relevant and important research, especially since articles related to COVID-19 are still being published. This is a significant drawback for review articles, which should be published in a timely manner to maintain their value. Another option is to extend the search and re-write the article.

**Author Response: As mentioned in the methods section, the review included a search of the global literature database on COVID-19 and lifestyles, initially in September 2020, and updated the search in PubMed on 31 January 2021. Hence, the review included all studies from the onset of the pandemic until 31 January 2021. To ensure timely publication, the review was immediately published in F1000 in the same year. Identifying peer reviewers took time, and with the peer review complete now, addressing the reviewers' comments in a satisfactory manner will help index the article. This will help greater reach and can inspire further research. Given the narrative nature of the article, the evidence generated should be sufficient in the humble view of the authors for other researchers to carry out more robust reviews such as systematic reviews and metanalysis on the impact of COVID-19 related lockdown on each of the six lifestyle factors. **

1. The researchers removed papers unrelated to COVID-19. As previously noted, if the issue is lockdown during a transmissible disease, the other papers should have been added. Otherwise, terminology and expressions throughout the document should be specific to COVID-19.

**Author Response: The authors confirm that they have maintained language specific to COVID-19 throughout the manuscript. **

1. Why were articles that examined the same factors among healthcare workers excluded? What do researchers mean when they state "additional stressors beyond the effects of the lockdown measures"? If they are more susceptible to contracting the virus, delivery and transportation workers are also at risk. If it is anything else, please provide a reasonable explanation as to why the pressures of other professions, in addition to the consequences of the lockdown, are not regarded significant enough to eliminate from the search. The review's goal is to examine how people's lifestyles changed during the pandemic. I don't see why the researchers made such a difference.

**Author Response: The reasons for exclusion have now been elaborated in the manuscript by highlighting the additional stressors faced by health workers in the methods section. These additional stressors make them a very specific group affected by COVID-19. The review focusses only on general population and restricts itself to age, income and gender differentials where applicable.**

1. The researchers created statements by merging articles from various geographical and cultural backgrounds. Dietary choices and physical exercise, for example, can vary. What is normal in one culture may be unhealthy in another, therefore the changes observed during the lockdown are not proportional.  I'm not sure if such a global mix of specific lifestyle behaviors is fair for comparison.

**Author Response: This limitation of the study is now made explicit under the limitations section.**

# Reviewer report: Reviewer response for version 1

**DOI:** 10.5256/f1000research.55830.r291274

**Published:** 2024-06-29

**Authors:** Anna Williams

**Affiliations:** 1 Save the Children Bangladesh, Dhaka, Dhaka Division, Bangladesh

The article describes the global literature on how people's patterns of behavior changed during COVID-19 lockdowns in regard to diet, physical activity, sleep, and substance use, and the effects on stress, emotional wellbeing and non-communicable diseases. It provides a narrative review with a critical interpretive synthesis and recommends increased public health investment and efforts around lifestyle medicine, or disease prevention through healthy lifestyle habits.

 The article is well-written and presents a comprehensive analysis.

 My largest comments are:

 1) The timeframe of the 'lockdown period' is not explicitly defined. Across countries, lockdowns were shaped by national and sub-national policies, and likely varied substantially in their implementation. It would be helpful if, at minimum, a defined lockdown period of time that the search focused on were stated. Some reflection on how lockdown timeframes and policies differed across and within countries would contribute helpful nuance to the overall analysis.

 2) The use of the present perfect tense (e.g., has had...has encouraged etc.) instead of the past tense makes it feel as if lockdowns are still happening, when at this point, they are now in the past. Suggest revising to consistently use the past tense.

 More minor comments:

 3) In Dietary Habits, Galican should be Galician.

 4) Recommend that consideration be given, in the mention of families not engaging in meditation and yoga at home, to that families often destress through joint leisure activities, such as playing games and doing projects together.

Is the work clearly and accurately presented and does it cite the current literature?

Partly

If applicable, is the statistical analysis and its interpretation appropriate?

Not applicable

Are all the source data underlying the results available to ensure full reproducibility?

No source data required

Is the study design appropriate and is the work technically sound?

Yes

Are the conclusions drawn adequately supported by the results?

Yes

Are sufficient details of methods and analysis provided to allow replication by others?

Yes

Reviewer Expertise:

Medical and health publications, plain language writing, public health, maternal and newborn health, midwifery

I confirm that I have read this submission and believe that I have an appropriate level of expertise to confirm that it is of an acceptable scientific standard, however I have significant reservations, as outlined above.

# Response

**Published:** 2024-09-22

**Authors:** Sathyanarayanan Doraiswamy

The authors would like to thank the reviewer for the comments. Our point-by-point response is as below:

 1) The timeframe of the 'lockdown period' is not explicitly defined. Across countries, lockdowns were shaped by national and sub-national policies, and likely varied substantially in their implementation. It would be helpful if, at minimum, a defined lockdown period of time that the search focused on were stated. Some reflection on how lockdown timeframes and policies differed across and within countries would contribute helpful nuance to the overall analysis.

**Lockdown timeframes are now included in the introduction section.**

 2) The use of the present perfect tense (e.g., has had...has encouraged etc.) instead of the past tense makes it feel as if lockdowns are still happening, when at this point, they are now in the past. Suggest revising to consistently use the past tense.

**Tense has been corrected to past tense in all applicable places.**

 More minor comments:

 3) In Dietary Habits, Galican should be Galician.

**This is now corrected.**

 4) Recommend that consideration be given, in the mention of families not engaging in meditation and yoga at home, to that families often destress through joint leisure activities, such as playing games and doing projects together.

**This is now included in the sub-section on emotional well-being and social connectedness.**

# Reviewer report: Reviewer response for version 1

**DOI:** 10.5256/f1000research.55830.r127652

**Published:** 2024-06-26

**Authors:** Magdalena Górnicka

**Affiliations:** 1 Department of Human Nutrition, Warsaw, Poland

This narrative review focuses on the impact of COVID-19 lockdown on six lifestyle factors: diet, physical activity, sleep, stress, social connectedness, and the use of tobacco, alcohol, or other harmful substances. The authors collected a significant number of articles, closely related to the title and the aim.

 I consider the description of the methodology for including articles in the review to be sufficiently described.

 I have one major comment:

- In the subsection Dietary habits, the authors also took into account the results concerning changes in body weight, which are a result / consequence of nutrition but not a lifestyle factor. In my opinion, this subsection requires modification and showing changes in dietary habits. Conclusions should take into account changes in body weight, which were also mainly conditioned by decreased activity.

Is the work clearly and accurately presented and does it cite the current literature?

Yes

If applicable, is the statistical analysis and its interpretation appropriate?

Not applicable

Are all the source data underlying the results available to ensure full reproducibility?

Yes

Is the study design appropriate and is the work technically sound?

Yes

Are the conclusions drawn adequately supported by the results?

Yes

Are sufficient details of methods and analysis provided to allow replication by others?

Yes

Reviewer Expertise:

nutrition, nutritional assessment, dietary habits

I confirm that I have read this submission and believe that I have an appropriate level of expertise to confirm that it is of an acceptable scientific standard, however I have significant reservations, as outlined above.

# Response

**Published:** 2024-09-22

**Authors:** Sathyanarayanan Doraiswamy

The authors would like to thank the reviewer for the comments. Our point-by-point response is as below:

 In the subsection Dietary habits, the authors also took into account the results concerning changes in body weight, which are a result / consequence of nutrition but not a lifestyle factor. In my opinion, this subsection requires modification and showing changes in dietary habits. Conclusions should take into account changes in body weight, which were also mainly conditioned by decreased activity.

 Response: The subsection on dietary habits has now been reorganized to distinguish between habits and consequences.

# Reviewer report: Reviewer response for version 1

**DOI:** 10.5256/f1000research.55830.r115298

**Published:** 2022-01-06

**Authors:** Ahmed Shihab Albahri

**Affiliations:** 1 Faculty of Human Development, Sultan Idris University of Education (UPSI), Tanjong Malim, Malaysia

Dear authors

 My comments can be explained below: 

1. Remove references from the conclusion section. 
2. There is no clear conclusion that has been raised in this manuscript. Provide more criticism in the abstract, introduction, and conclusion. 
3. Why do authors depend their search based on two databases only? What about other reliable databases such as Web of Science, Science Direct, and Scopus? I think there is no justification for this concern. 
4. How can narrative review include a method like a systematic review? What is the difference? The authors should report that to the readers. 
5. I can see the introduction is not suitable when it is presented. The introduction should clarify many subjects within. Thus, the introduction needs to clarify the motivation, challenges, contribution, objective, significance, and others. All the information should be presented in sequence idea. 
6. Write the conclusion and consider the following comments: Highlight your analysis and reflect only the important points for the whole paper. Mention the benefits.  Mention the implication in the last of this section.
7. The authors mentioned, "Articles were excluded if they did not focus specifically on the six lifestyle factors (dietary habits, physical activity, sleep, tobacco or other substance use, stress, and emotional well-being and social connectedness) or did not discuss lifestyle within the COVID-19 context." Why? what about others factors. Normally, this type of review is a comprehensive, critical, and objective analysis of the current knowledge on a topic. While we see here, the authors specified these factors from the beginning. I need the authors to defend this comment in the revised manuscript within the "method" section. 
8. I am surprised the introduction contains only one reference. Also, there is no comparison with any published review articles. Therefore, the authors should provide a clear comparison with academic literature based on specific points. At least four published articles should be included in the benchmark comparison. I added some citations that the authors could use in this comparison. 

In conclusion, I see this manuscript is sound and a high-quality study *.*

*Best Wishes*

Is the work clearly and accurately presented and does it cite the current literature?

Yes

If applicable, is the statistical analysis and its interpretation appropriate?

Yes

Are all the source data underlying the results available to ensure full reproducibility?

Yes

Is the study design appropriate and is the work technically sound?

Yes

Are the conclusions drawn adequately supported by the results?

No

Are sufficient details of methods and analysis provided to allow replication by others?

Partly

Reviewer Expertise:

medical informatics

I confirm that I have read this submission and believe that I have an appropriate level of expertise to confirm that it is of an acceptable scientific standard, however I have significant reservations, as outlined above.

## References

[1] : COVID-19 lockdown impact on lifestyle habits of Italian adults. *ActaBiomed* .2020; 10.23750/abm.v91i9-S.10122 10.23750/abm.v91i9-S.10122 PMC802309632701921

[2] : Role of biological Data Mining and Machine Learning Techniques in Detecting and Diagnosing the Novel Coronavirus (COVID-19): A Systematic Review. *J Med Syst* .2020;44(7) : 10.1007/s10916-020-01582-x 122 10.1007/s10916-020-01582-x 32451808 PMC7247866

[3] : The Effects of COVID-19 and Quarantine Measures on the Lifestyles and Mental Health of People Over 60 at Increased Risk of Dementia. *Front Psychiatry* .2020;11: 10.3389/fpsyt.2020.578628 578628 10.3389/fpsyt.2020.578628 33173523 PMC7591702

[4] : Systematic review of artificial intelligence techniques in the detection and classification of COVID-19 medical images in terms of evaluation and benchmarking: Taxonomy analysis, challenges, future solutions and methodological aspects. *J Infect Public Health* .2020;13(10) : 10.1016/j.jiph.2020.06.028 1381-1396 10.1016/j.jiph.2020.06.028 32646771 PMC7328559

# Response

**Published:** 2024-09-22

**Authors:** Sathyanarayanan Doraiswamy

**The authors would like to thank the reviewer for the comments. Our point-by-point response is as below:**

 1.         Remove references from the conclusion section.

**This has now been done.**

 2.         There is no clear conclusion that has been raised in this manuscript. Provide more criticism in the abstract, introduction, and conclusion.

**The conclusion has been revised based on the valuable suggestions provided by the reviewer.**

 3.         Why do authors depend their search based on two databases only? What about other reliable databases such as Web of Science, Science Direct, and Scopus? I think there is no justification for this concern.

**Justification now provided in the methods section.**

 4.         How can narrative review include a method like a systematic review? What is the difference? The authors should report that to the readers.

**Key difference highlighted and a reference for further reading included in the introduction section.**

 5.         I can see the introduction is not suitable when it is presented. The introduction should clarify many subjects within. Thus, the introduction needs to clarify the motivation, challenges, contribution, objective, significance, and others. All the information should be presented in sequence idea. **The introduction is presented in sequence in tandem with comments from all reviewers.**

 6.         Write the conclusion and consider the following comments: Highlight your analysis and reflect only the important points for the whole paper. Mention the benefits.  Mention the implication in the last of this section.

**The first part of the conclusion has been rewritten in accordance with the reviewer’s kind suggestion.**

 7.         The authors mentioned, "Articles were excluded if they did not focus specifically on the six lifestyle factors (dietary habits, physical activity, sleep, tobacco or other substance use, stress, and emotional well-being and social connectedness) or did not discuss lifestyle within the COVID-19 context." Why? what about others factors. Normally, this type of review is a comprehensive, critical, and objective analysis of the current knowledge on a topic. While we see here, the authors specified these factors from the beginning. I need the authors to defend this comment in the revised manuscript within the "method" section.

**The review looks at six evidence-based lifestyle factors identified by the American College of Lifestyle Medicine. This is mentioned in the introduction and re-emphasized in the methods section.**

 8.         I am surprised the introduction contains only one reference. Also, there is no comparison with any published review articles. Therefore, the authors should provide a clear comparison with academic literature based on specific points. At least four published articles should be included in the benchmark comparison. I added some citations that the authors could use in this comparison.

**Four articles are now included in the introduction section. The specific articles kindly identified by the reviewer have also been included in the introduction and discussion sections.**
